# Genetic View on the Phenomenon of Combined Diseases in Man

**Published:** 2009-10

**Authors:** V.P. Puzyrev, M.B. Freidin

**Affiliations:** 1 Research Institute for Medical Genetics, Siberian Branch, Russian Academy of Medical Sciences

## Abstract

In clinical medicine, the phenomenon of polypathy, as a particular object of investigation, was first put forth by French clinicians at the end of the 19th century through the "arthritismus" doctrine. In the first half of the 20th century, German paediatricians singled out "syntropias," which are combinations of diseases with common pathophysiological mechanisms, and "dystropias," which are diseases that rarely co-occur in one individual. In the present paper, syntropy/dystropy is defined as a natural generic nonrandom phenomenon with an evolutionary-genetic basis. The genes involved in the development of syntropy are called "syntropic genes," whereas the genes that co-participate in pathophysiological mechanisms and prevent the co-occurrence of particular phenotypes are called "dystropic genes." Prospects for studying the genetic basis of this phenomenon are highlighted. The publicly available database HuGENet can be used in order to identify syntropic genes, as will be shown as examples in an analysis of cardiovascular diseases.

## INTRODUCTION

Global epidemiological studies of human diseases have yielded plenty of results, among which three observations deserve special attention when considering polypathy and the phenomenon of polymorbidity; that is, the situation in which an individual carries several diseases at the same time. First, only 30 chronic multifactor diseases account for 65 % of all the diseases human beings suffer from, accounting for morbidity and mortality rates in contemporary populations [1]. The risk of contracting such a multifactor disease in one's lifetime is estimated at 60 % [2] in Western populations. Secondly, polypathy is typical of the clinical state of a contemporary patient. In patients over 65 years of age, it is common to observe more than ten related diseases in clinical practice; the co-occurrence of diseases in women is more frequent than in men (this is true for all age groups) [3]. Finally, genetic epidemiology studies of common multifactor diseases point to the importance of inherited factors in their appearance and development. The role of genetic or inherited factors in determining the common phenotype of different diseases can vary, but even with low heritability indices (h^2^ = 20-30 %), the genetic factors that affect vulnerability to infectious agents such as viruses, bacteria, helminths and parasites can be determined [4]. 

## COMBINED DISEASES: SYNTROPY VS. DYSTROPY

The term «polypathy» refers to any manner of combinations of diseases and syndromes in a single patient, including accidental maladies (traumas, iatrogenic illness, etc.). However, special forms of polypathy do exist and were combined under the term "syntropy" offered by German paediatricians M. Pfaundler and L. von Seht. They defined syntropy as the "mutual disposition, attraction" of two disease conditions, whereas they refer to "mutual repulsion" as dystropy [5]. In the same authors' opinion, a "syndrome" can also be regarded as syntropy, because it includes selective affinity of the traits it is made of. Another feature of the affinity of pathological conditions is synchrony, the appearance of at least two diseases simultaneously. 

As proof that the syntropy concept is relevant, Pfaundler and von Seht collected and analysed about 30 thousand medical records for children starting from 1906. They offered an index of syntropy (S) showing how much the observed number of combinations of the diseases differs from the number that would be expected at random and provided many examples of syntropias with a high S index. For instance, "congenital heart disease – joint rheumatism" (S = 58.55), "psychopathy – enuresis" (S = 15.31), "hyperthyrosis – nephropathy" (S = 4.94), and "nephritis – exudative erythema" (S = 4.49). 

Recently, a number of papers have been published whereby a similar idea was tested and proven using contemporary, sophisticated statistical methods [6-8]. They clearly demonstrated a significantly more or significantly less frequent co-occurrence of certain diseases than would be expected at random. 

Actually, interest in polypathy was expressed even earlier, in the 19th century, through the concept of "arthritismus" by French clinician Charles Bouchard, who defined arthritismus as a specific disposition toward a group of diseases occurring either in isolation or in different combinations in an individual or among many members of his or her family [9]. In the opinion of the author of the concept, these diseases had a common basic effect on metabolism: namely, they decreased it (bradytrophy). 

Along with syntropic interaction between diseases, antagonistic relationships, so-called "dystrophic" diseases, are described. Examples of dystropic diseases include lung tuberculosis and mitral stenosis, type I diabetes mellitus and peptic ulcer disease, lymphoproliferative and myeloproliferative processes [3], as well as lung tuberculosis and bronchial asthma [9]. 

There are many well-known syntropic diseases, including cardiovascular diseases [10, 11]; immune-mediated diseases (allergic diseases, autoimmune diseases) [12-14]; endocrine diseases including the combination of diabetes mellitus, autoimmune thyroiditis, and celiac disease [15, 16]; and psychiatric diseases, such as major depressive disorders and bipolar disorder [17], narcotic and addictive states [18, 19]. 

To assess the genetic contribution to syntropy, it is important to return to its definition: it is the natural generic nonrandom phenomenon of combination of two or more pathological states in an individual and his or her nearest relatives, with an evolutionary and genetic basis [20]. Syntropias comprise just part of all known polypathias. They include etiologically and pathogenetically linked combinations of diseases, unlike random combinations.

Nonrandom combinations of pathologies in an individual and his or her relatives can highlight the common genes involved in the disposition to separate diseases. Genetic studies of multifactor diseases strengthen our confidence that there are common genetic roots to such combinations (syntropias), notably when such combinations occur more often in families of patients with these groups of diseases compared to the general population. 

In this aspect, we postulated that against the background of the huge number of human phenome characteristics one can carve out a fairly legible sector including a considerable number of interrelated pathological traits – syntropias – for which genetics is a worthy subject of investigation. Genes corresponding to such syntropic traits are called syntropic genes [20].

## SYNTROPIC GENES (a GENERAL VIEW ON THE PROBLEM)

Epidemiological studies of complex diseases in humans provide good examples of syntropias (cardiovascular diseases continuum, allergic diseases, autoimmune diseases). Yet, for all syntropias it is important to identify the groups of genes that will determine one or the other pathophysiological pathways and can help predict the risk of syntropy among carriers of one or other combinations of those genes. Physical clustering of susceptibility genes in the human genome has been shown for a number of diseases and traits [21-23]. However, syntropic genes do not necessarily belong to a cluster of physically linked genes, but rather represent a set of functionally interacting genes dispersed throughout the human genome, co-regulated and involved in a common biochemical or physiological pathway. 

Autoimmune disorders were among the first groups of diseases studied from the point of view of common genetic determinants in their development. There are many common elements in the clinical phenotype of autoimmune disorders, approaches to their therapy, population prevalence, gender ratio (75% of patients with autoimmune diseases are females), and occurrence in families. Becker K.G. et al. put forward a hypothesis assuming that, in some cases, clinically different autoimmune diseases can be controlled by a common set of susceptibility genes [21]. They performed a comparison of the linkage results from 23 published autoimmune or immune-mediated disease genome-wide scans in humans (multiple sclerosis, Crohn's disease, psoriasis, asthma, and type I diabetes) and animals (experimental autoimmune encephalomyelitis, rat inflammatory arthritis, rat type I diabetes, murine type I diabetes, Bordetella pertussis-induced histamine sensitization, immunity to exogenous antigens, and murine system lupus erythematosus); non-autoimmune disorder genome scans were analyzed as well (type II diabetes, schizophrenia, bipolar disorder, leptin-dependent obesity, and hypertension). It was shown that the majority (about 65%) of human positive linkages for immune-mediated diseases map non-randomly into 18 distinct clusters and overlapping susceptibility loci occur between different human immune diseases. A similar pattern was observed in experimental autoimmune/immune disease models. A number of autoimmune candidate genetic loci from different diseases did not fall into identifiable clusters, and these singleton loci, in the author's opinion, may be independent loci; they may contribute to disease specific susceptibility, tissue or organ tropism, or may be false positives. In the control group of nonimmune-mediated human diseases, linkage with autoimmune/immune clusters was rare. 

The need to screen the «immunological genome» for detection of the genetic basis of infectious, inflammatory, and autoimmune diseases was pointed out in the early 1990s [24]. A similar issue was formulated by geneticists in respect to the long-standing clinical observation of the existence of another immunological syntropy including psoriasis, psoriatic arthritis, atopic dermatitis, and asthma. However, in the pre-genomic period, it was established that psoriasis is a clear example of a Th1-mediated disease (cellular immunity) controlled by IFN-γ gene expression, unlike the Th2-mediated diseases (humoral immunity), such as asthma, for which IL-4 gene expression is important. Genomic studies of these diseases confirmed this: asthma, at least partially, results from molecular-genetic mechanisms different from those involved in psoriasis. To date, multiple genome-wide linkage studies and a number of genome-wide association scans for asthma have been performed, and several genomic regions have been repeatedly identified, including those on chromosomes 2q33, 5q23-31, 6p24-21, 11q12-13, 12q24-12, and 13q14-12. Eight new asthma susceptibility genes have been discovered by positional cloning: ADAM33 (desintegrin and melloproteinase-33), DPP10 (dipeptidyl peptidase-10), PHF11 (plant homeodomain finger protein-11), GPRA (G protein-coupled receptor for asthma), HLA-G (histocompatibility antigen), CYFIP2 (cytoplasmic fragile X mental retardation-interacting protein 2), IRAKM (interleukin 1 receptor-associated kinase), and OPN3 (opsin-3) [25, 26]. It has been shown that asthma loci and atopic dermatitis loci indentified by genomic scans rarely overlap [12, 27]. At the same time, genome-wide linkage scans have identified multiple loci linked to atopic dermatitis and psoriasis and revealed shared susceptibility loci for these diseases on chromosomes 1q21, 3q21, 17q25 and 20p12 [28]. Hence, in the syntropy including four diseases assumed earlier, asthma genetically significantly differs from the other three disorders. 

Certainly, the abovementioned premises are just a general conception of syntropy and its genetic basis (syntropic and dystropic genes). The genetic component of this phenomenon, noticed earlier, should be the subject of contemporary studies based on advances in molecular biology and genetics, as well as functional genomics and bioinformatics. Such concepts help advance the complex problem of defining the genetic basis of common multifactorial human diseases. 

A similar disease network hypothesis was expressed and exploited in a recent study by Rzhetsky A. et al. [7]. They analysed 1.5 million patient records for 161 disorders and offered an approach that allowed to estimate the extent of genetic overlaps between the diseases. Based on the results, Rzhetsky A. et al. concluded that "disease phenotypes form a highly connected network of strong pairwise correlations" and speculated that this can be immediately applicable to genetic mapping studies involving multiple, apparently disparate phenotypes.

## syntropic genes for cardiovascular disease continuum

Based on the concepts and definitions presented above, we aimed to define syntropic genes for a well-known group of syntropic diseases – cardiovascular disease continuum (CDC), including coronary artery disease (CAD), arterial hypertension (AH), stroke, metabolic syndrome (MS), dislipidemia (DL), obesity, and noninsulin-dependent diabetes-mellitus (NIDDM).

The CDC concept was initially presented in 1991 [10]. This idea considers cardiovascular diseases (CVD) as a sequential line of events determined by multiple, related and unrelated risk factors, progressing over a number of physiological and metabolic pathways and resulting in the development of the final stage of a heart disease. The continuum members (diseases and traits) overlap and interact as a sequence of discrete and tandem states during the progression of CVD [10, 11]. This context allowed us to refer this unity of pathological conditions to a syntropy. 

We used the publicly available research tool HuGE Navigator to identify the genes underlying the development of seven CDC diseases (as specified above). The HuGE Navigator provides access to a continuously updated human genome epidemiology database that includes information on population prevalence of genetic variants, gene-disease associations, gene-gene and gene-environment interactions, and the evaluation of genetic tests. It is based on the achievements of the Human Genome Epidemiology Network (HuGENet™), a voluntary, international collaboration focused on assessing the role of human genome variation in health and disease at the population level. Since 2001, HuGENet™ has maintained a database of published, population-based epidemiologic studies of human genes extracted and curated from PubMed [29].

In the HuGENet database, the number of genes studied in relation to the seven CVDs varied between 162 for DL and 466 for AH. HuGE Navigator ranks these genes by score for a given gene estimated as the ratio of the number of studies showing positive results for an association to the total number of published studies.

To stress the strength of the associations between genes and diseases, we only considered genes with a score equal to or greater than 0.01. The maximum scores for different genes associated with the diseases analyzed were 4.1 for DL, 1.60 for CAD, 1.12 for AH, 1.02 for stroke, 1.01 for MS, 0.74 for obesity, and 0.36 for NIDDM.

Twenty-one of the genes found were associated with all of the CDC diseases (Table 1). Certainly, the number of genes underlying any particular disease included in CDC syntropy is much higher. However, for total CDC syntropy, in accordance with HuGENet data and ranking criteria, only these 21 genes can be attributed to the control of the development and structure of CDC syntropy itself, and these genes can be called syntropic genes of CDC. Two features merit special attention. First, most of these genes are well-characterized and have been studied at length. Secondly, they comprise the inherited basis of the pathophysiological continuum of mechanisms underlying the development of this syntropic disease group, including dysfunction in lipid metabolism, renin-angiotensin-aldosteron system, sympathoadrenal system, inflammation, and endothelial function. 

**Table 1:** Syntropic genes for cardiovascular disease continuum

Order number	Gene symbol	Gene product	Chromosomal localisation
1	*ABCA1*	ATP-binding cassette transporter 1	9q22-q21
2	*ACE*	Angiotensin I-converting enzyme	17q23
3	*ADIPOQ*	Adipocyte-specific secretory protein	3q27
4	*ADRB2*	β_2_-adrenergic receptor	5q32-q34
5	*AGT*	Angiotensinogen	1q42-q43
6	*AGTR1*	Angiotensin receptor 1	3q21-q25
7	*APOA1*	Apolyporpotein А1	11q23
8	*APOE*	Apolyporpotein Е	19q13.2
9	*CETP*	Cholesteryl ester transfer protein	16q21
10	*ESR1*	Estrogen receptor 1	6q25.1
11	*GNB3*	Beta-3 G-binding protein	12p13
12	*IL6*	Interleukin-6	7p21
13	*LIPC*	Hepatice lipase C	15q21-q23
14	*LPL*	Lipoprotein lipase	8p22
15	*LTA*	Lymphotoxin-α	6p21.3
16	*MTHFR*	Methylenetetrahydrofolate reductase	1p36.3
17	*NOS3*	Endotelia NO-synthase	7q36
18	*PPARG*	Peroxisome proliferator-activated receptor-γ	3p25
19	*SERPINE1*	Plasminogen activator inhibitor 1	7q21.3-q22
20	*SELE*	Selectin E	1q23-q25
21	*TNF*	Tumor necrosis factor-α	6p21.3

The results of genetic association studies (GAS) are controversial due to sample heterogeneity (ethnic variety, age and gender differences), relatively small sample sizes (mega-studies have appeared only recently), and indistinct clinical criteria in disease group recruiting. Taking this into account, it is generally assumed that GASs should be accompanied by meta-analysis, as well as genome-wide association studies (GWASs). These principles were taken into account in the development of HuGENet, and genes appearing in GWAS and meta-analysis receive higher coefficients.

Ninety-one meta-analysis studies carried out for 21 genes and seven diseases were found in HuGENet. The numbers vary for different diseases, and the maximum values found are for the MTHFR and APOE genes (18 and 13 studies, respectively). No meta-analyses have been published so far for the SELE, ESR1, and SERPINE1 genes. Among the diseases considered, CAD, stroke, AH, and obesity were the most common subjects of meta-analysis: 28, 21, 17, and 13 studies, respectively.

At the time of this study, in the HuGENet, there were 13 meta-analyses for the APOE gene, and CAD and stroke. Recent analyses [30] included 203 studies for a period from 1970 to 2007, providing ultimate proof of the significant association between genetic variants of the APOE gene and CAD and stroke. For carriers of the E4 allele, the risk of CAD development is 20% higher than those without it, and the cholesterol levels in low-density lipoproteins increase in the following direction (in terms of the presence of the APOE allele): E2/E2, E2/E3, E2/E4, E3/E3, E3/E4, E4/E4. The odds ratio (OR) for CAD development in carriers of E4 is 1.06 (95% CI 0.99-1.13). In a review of 500 papers [31], a significant and direct association between the APOE and strokes in Asians (Chinese, Japanese, Koreans)---but not in Caucasians---was established. Also, the association of strokes with other genes (ACE I/D, MTHFR 677C/T) was confirmed. Thus, three phenotypes of the CDC syntropy express association with APOE and the association is confirmed by meta-analyses.

For the other gene subjected to numerous meta-analyses, MTHFR, the association with five CDC phenotypes (CAD, AH, stroke, NIDDM, obesity) and its C677T polymorphism is rather supported [32-36] than not [37]. It is notable that the MTHFR polymorphism is an independent risk factor for AH [38].

The association of the ACE gene with CVD has been studied for a long period of time, and a large body of data has been accumulated. However, meta-analyses for the gene and their disease groups have been initiated only recently. There are seven such publications in the HuGENet database. In the meta-analysis of 118 studies, the I/D polymorphism of ACE was conclusively shown to be associated with CAD and NIDDM [39]; however, there are meta-analyses that cannot confirm this association [36, 40]. Meta-analysis of the other gene in the rennin-angiotensinogene system, AGT, showed significant association between its polymorphism M235T and CAD, AH, obesity, and stroke; in all cases, the 235T/T genotype is associated with increased risk of diseases [41, 42].

For the association of the LPL gene and CVDs, the results of meta-analyses are quite controversial. There are just seven such analyses, and they deal with separate multiple polymorphisms of the gene. The results of one meta-analysis study identified an association between the Asn291Ser mutation and CAD, NIDDM, and DL (hypertrigliceridemia and low level of cholesterol in high-density lipoproteins) [43]. A meta-analysis in which, for the first time, a gene-gene interaction between APOE and LPL was shown has been published [44]. According to this study, in co-carriers of АРОЕ* Е4 and LPL*447X (S447X polymorphism), OR for stroke and myocardial infarction development was 2.2 (р = 0.01).

Three genes critical for the development of inflammation were shown to be important for CDC syntropy: IL6, TNF, and LTA. For the LTA gene, only one meta-analysis is known (for CAD); however, for the IL6 and TNF genes, five and seven meta-studies are cited in HuGENet, respectively. IL6 was examined in meta-analyses for association with NIDDM and CAD and confirmed the lack of any association [45-47]. For the TNF gene, there are meta-analyses for all diseases involved in the CDC syntropy, except for DL. The analysis of 31 studies of MS and -308G/A polymorphism of TNF showed that -308A allele carriers show a 23% higher risk of obesity; they also have significantly increased systolic blood pressure and plasma insulin levels [48]. The same polymorphism shows stable association with CAD and stroke in Asians: genotype -308G/G carriers show a 40% lower risk of stroke than the others [49].

For the other CDC syntropic genes and phenotypes, there are very few meta-analyses, and it is important to study them further using this method. 

**Fig. 1.**
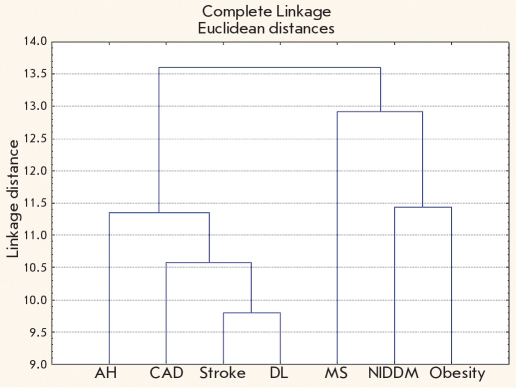
Tree diagram showing clusters of cardiovascular disease continuum members based on the number of shared/non-shared genes associated with them. MS – metabolic syndrome; NIDDM – non-insulin dependent diabetes-mellitus; AH – arterial hypertension; CAD – coronary artery disease; DL – dislipidemia

Based on the information on shared and non-shared genes, we conducted a hierarchical cluster analysis of the CDC syntropy diseases to determine whether they have gene-based relationships. Two tight clusters are seen; one is comprised of AH, CAD, stroke and DL, while the other is composed of MS, obesity, and NIDDM (Fig. 1). 

It seems reasonable that AH, CAD, stroke, and DL form a close cluster by shared susceptibility genes. This has been proven by Dzau V. et al. [11] in their paper presenting the CDC concept. For these four components of CDC, the overlap of basic pathways (lipid metabolism, rennin-angiotensin-aldosterone and adrenergic systems, oxidative stress, and endothelial dysfunction) in the phenotype development is demonstrated [34, 41].

However, MS, obesity, and NIDDM have their common and specific features in pathogenesis. The common feature being that their leading pathway is insulin metabolism. Nevertheless, MS is distinguished by resistance to hyperinsulinism and insulin, while NIDDM is characterized by the impaired pancreatic beta-cell function and insulin resistance [50, 51]. These features can be attributed to the relations between MS, NIDDM, obesity, and the other CVDs studied, which are reflected in the cluster diagram.

The approach presented here can potentially be applied to any other syntropic disease group. Presumably, it could be interesting to analyse the genetic clustering of the plethora of human diseases for the purpose of building a generic system for their classification. 

A similar approach was used recently by Torkamani A. et al. [8], who showed a high correlation between the SNPs significant in the GWAS for CAD, AH, and NIDDM, as well as for biporal disorder (BD) and a number of immune-mediated diseases. In particular, they showed that among the top-ranked (in terms of the statistical significance of their association with diseases) 1 000 SNPs, there are 57 shared by CAD and NIDDM, 81 shared by AH and NIDDM, and 63 shared by AH and CAD. These genetic correlations between the diseases were highly significant. Also, strong correlation between the autoimmunity-related disorders rheumatoid arthritis and insulin-dependent diabetes-mellitus was demonstrated. Surprisingly, a strong genetic correlation of BD and CAD and NIDDM, as well as a strong correlation between AH and Crohn's disease – seemingly unrelated diseases – was observed. This suggests some unexpected links between these diseases and argues for the utility of a genetic correlation-based approach for natural disease categorization.

## Conclusion

"Phenome" by analogy with the term "genome" is defined as the exact phenotypic representation of a species [52]. It includes the morphological, biochemical, physiological, and ontogenetic characteristics of an organism. Phenomics seeks to define the extent of variability in the phenome but represents a major challenge. The view of a pathological phenotype from the point of view of nonrandom combination of morbid traits (syntropy) does not coincide with the clinical tradition of paying primary attention to a particular diagnosis, a nosology. In the syntropic approach, from the infinite number of traits of the phenome, a sampling of interrelated traits controlled by common genes is assumed. The way to identify such syntropic genes is not significantly different from what is done for the genetic analysis of any non-Mendelian trait. However, substantially larger population sample sizes will be required to achieve confidence in a gene-phene link. Moreover, unification (standardisation) of a phenotype is critical, if very time-consuming, and dependent on the clinicians and epidemiologists involved in ongoing epidemiological studies in different regions of the world. 

In this respect, it is also worth noting the very well-recognized phenomenon of pleiotropy, the multiple phenotypic effects of a single gene. A few recent studies discuss this problem in application to human diseases and put forth ideas similar to the concept of syntropy [53, 54]. Likely, pleiotropy is one of the basic factors of syntropy development. The sum of the pleiotropic effects of genes constitutes the physiological fields of their action, which can be described as the gene's network or, in a more common sense, as the biological network. The overlap of the fields of action of the genes forms a meta-field, which is the basis for the development of a group of diseases bearing relation to these genes. Given the common genetic background based on the interaction between a limited set of genes, these diseases would have a tendency to cluster together, constituting a syntropy. At the same time, these diseases are phenotypically distinguishable, because different parts of the gene meta-field of action will have a conclusive significance for different diseases.

An increase in genetic association studies, both candidate-gene-based and GWAS-based, is forecasted [39]. This prognosis is based on the advances in the availability of mapped SNPs, finalisation of the HapMap Project, microarray-based genotyping technology development, and evolution of statistical and bioinformatical methods. However, looking for genetic markers for complex disease risk is not as straightforward as detecting phenotypic biomarkers of the disease risk currently used in clinical practice [55].

Actually, the OR attributable to most alleles associated with complex diseases both in GASs and GWASs rarely reaches a value of 1.15-1.50; usually, even weaker associations are detected, and their application to clinical practice is estimated to be low [55]. However, it cannot be excluded that the weak effect is a consequence of the genetic and phenotypic heterogeneity of the studied population, when there are individuals showing higher effects of respective genes, too. Taking this into consideration, it is suggested that a well-focused approach to the organization of the study will help detect stronger and more robust associations of alleles with pathological phenotypes, for instance, in young patients, among persons with a definite family aggregation of the diseases, or in patients frequently hospitalized [56]. In our opinion, the analysis of common genes for a chosen syntropy offered an option for such a well-focused study, which is helpful in discovering genes with strong effects and ranking them by their effect in relation to a pathophysiological continuum. 

## Acknowledgements

This work was supported in part by the Russian Foundation for Basic Research, (grants 07-04-01613, 07-04-01526, 08-04-01814).
